# Laparoendoscopic Management of Colonic Trichobezoar Causing Acute Large Bowel Obstruction in an Adolescent Girl

**DOI:** 10.1097/PG9.0000000000000064

**Published:** 2021-04-12

**Authors:** Riccardo Guanà, Luca Lonati, Fabio Cisarò, Antonio Pizzol, Salvatore Garofalo, Federico Scottoni, Alessandro Pane, Lucia Marocco, Fabrizio Gennari

**Affiliations:** From the * Pediatric General Surgery Unit, Regina Margherita Children’s Hospital, University Hospital of Health and Science, Turin, Italy; † Pediatric Gastroenterology Unit, Regina Margherita Children’s Hospital, University Hospital of Health and Science, Torino, Italy; ‡ Pediatrics Unit, Department of Pediatrics and Public Health, Turin University, Turin, Italy.

## INTRODUCTION

A bezoar is the accumulation of undigested foreign bodies or nutrients in the gastrointestinal tract, forming a conglomeration. Bezoars are named according to the accumulating materials: phytobezoar (fibers or seeds from vegetables or fruits), trichobezoar (hair), lactobezoar (remnants of milk), and lithobezoar (rocks or similar substances) (1). Phytobezoars are the most common bezoars found in children, being the stomach the most common site (2).

Trichobezoars usually occur in young girls and are related to the ingestion of their own hair. Psychiatric disturbance, behavioral disorders, mental retardation, parental separation, or abuse are reported in 20%–30% of patients affected by trichotillomania. In case of repeated trichophagia, the accumulated hair are mainly retained in the gastric cavity and the trichobezoar, in addition to entrapped food particles and mucus, impairs the gastric emptying through the pylorus. The majority of gastric trichobezoars present with abdominal pain and a palpable mass. Occasionally there can be extension of a long tail of hair into the small intestine, this condition, known as Rapunzel syndrome, and presents with small bowel obstruction symptoms, that typically reaches the terminal ileum (3).

Primary colonic obstruction due to a trichobezoar is also possible, but rare.

In case of bowel obstruction, surgical exploration and removal of bezoar are usually required (2).

If left undiagnosed, acute bowel obstruction occurs in 26% of patients and peritonitis from visceral perforation affects 18% (3).

Diagnosis is not easy, as patients with trichobezoars are usually asymptomatic until the mass is large enough to cause symptoms (abdominal pain, vomits, inability to pass stool, up to anemia, and malnutrition). In case of colonic bezoars, the first diagnosis that arises is that of a constipation or stool retention, and frequently, a series of abdominal X-rays are obtained, without directly evidencing the bezoar. In these cases, to collect an accurate medical history become crucial. If addiction to plain films, ultrasonography or computed tomography can provide more precise informations.

Recently, in our Pediatric Surgery Unit, we treated laparo-endoscopically a large bowel trichobezoar, which caused colonic obstruction in a 13-years-old girl with trichotillomania.

This is, to our knowledge, the first-ever reported case of a giant colonic trichobezoar with near-total colonic obstruction.

## PATIENT AND METHODS

**FIGURE 1.**
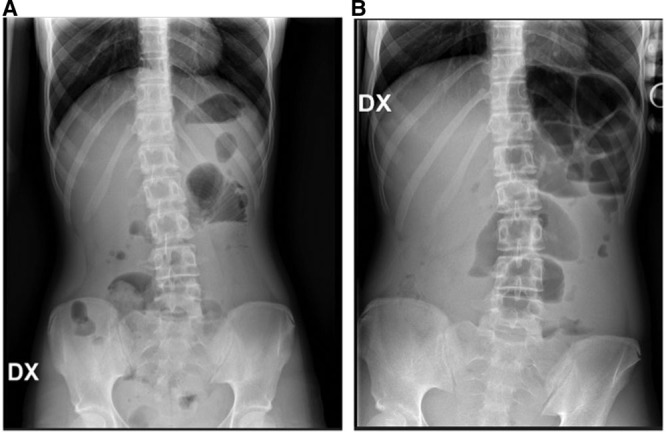
A) Plain abdominal x-rays showing bowel obstruction: air-fluid levels at the left upper quadrant with colonic gas distension. B) Worsening abdominal occlusion demonstrated by increased air-fluid levels.

A 13-year-old girl was admitted to our Emergency Department in January 2020 with abdominal pain and distention and vomiting. Clinical history was positive for chronic constipation exacerbated over the previous week; in fact, she did not pass stool in the previous 7 days. The family denied psychological disorders of the patient or trichotillomania. On examination, the abdomen was tense and painful, there were no palpable masses, possibly due to the adult-like size of the patient; per rectal examination was not performed; the laboratory tests were within the normal ranges. An abdominal X-ray was performed showing air-fluid levels in the left upper quadrant and colonic gas distension (Fig. 1). The patient also underwent an abdominal ultrasound, which did not reveal any abnormality. The initial approach was based on conservative treatment of an acute exacerbation of chronic constipation therefore, a naso-gatric tube was placed, to decompress the stomach, and a peripheral venous access inserted for fluid maintenance while fasting. Rectal irrigations were unsuccessfully performed. The patient underwent serial X-rays over the following 5 days, however, because of the lack of clinical improvement, and in presence of persistent abdominal distension, pain, and high naso-gatric aspirates, decision was taken to perform an explorative laparoscopy as a simultaneous diagnostic and possibly therapeutic approach. The procedure was planned with an available endoscopist to be ready to perform a colonoscopy in case of needing.

**FIGURE 2.**
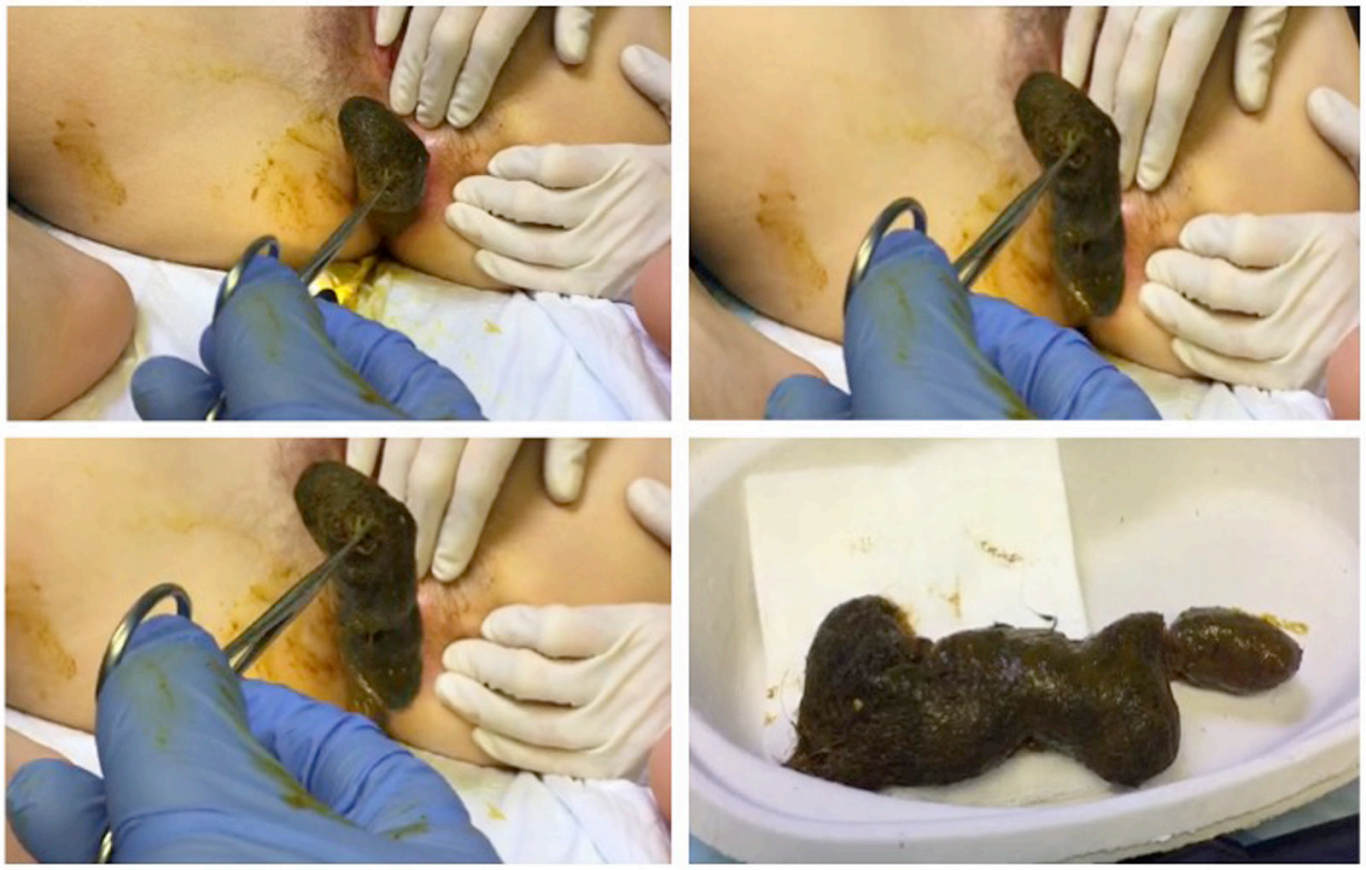
Macroscopic appearance of the bezoar.

The patient underwent a diagnostic laparoscopy, performed by inserting three 5mm trocars (transumbilical, left iliac fossa, and suprapubic, respectively) using a 5 mm 0° laparoscope. The exploration revealed a markedly distended ileum and colon, up to the rectosigmoid junction, where an evident caliber leap was present. A nonspecific and diffuse inflammatory status of the serosal surface of the colon and of the terminal ileum was found. Suspecting an intrinsic obstruction we proceeded to a colonoscopy. A giant trichobezoar was identified exactly at the level of the recto-sigmoid junction; endoscopic extraction was successfully attempted using a standard polypectomy snare to mobilize the conglomeration, the final extraction was achieved after a profuse colonic washout (Fig. 2). Luminal integrity was confirmed with rectosigmoidoscopy and the procedure was ended. The patient recovered well, and she was discharged the day after the procedure with an ongoing neuropsychiatric referral.

Informed consent was obtained from all individual participants (children’s parents) included in the study. Ethical approval: all procedures performed in this study were in accordance with the ethical standards of the Institutional and National Research Committee and with the 1964 Helsinki declaration and its later amendments or comparable ethical standards.

## DISCUSSION

Surgical and endoscopic treatment of gastric bezoars is well reported in the literature while, to our knowledge, this is the first report of an endoscopic extraction of a colonic trichobezoar within pediatric population. This is probably because trichobezoars are rarely found in the large bowel, being their main portion usually located in the stomach with, eventually, a tale extending to the small bowel or event ascending colon (Rapunzel Syndrome) (2,3).

Trichobezoars are usually asymptomatic however the progressive increase in size over the time, will lead to the development of bowel obstruction. Serious complications have been described such as gastric ulcers, perforation, or even death (4). A recent series reported that: Rapunzel syndrome accounting for 54% of the studied population; Gastric trichobezoar was found within 35% while small bowel trichobezoars were found within the 12% of the considered population. One patient, presented with massive rectal bleeding and gastric perforation, succumbed postoperatively; 1 patient developed a recurrent trichobezoar (5). A diagnostic algorithm for trichobezoars should include: (1) clinical and physical examination findings; (2) abdominal X-rays; (3) abdominal ultrasound; (4) abdominal CT/MR or explorative laparoscopy (the latter, despite more invasive, when performed as a combined procedure with endoscopy, offers the advantage to be a simultaneous diagnostic and therapeutic procedure); (5) contrast enema; (6) colonoscopy; (7) laparotomy/laparoscopy for enterotomy and bezoar removal.

Conventional laparotomy is traditionally the treatment of choice for large trichobezoars. The laparoscopic approach offers the advantages of precisely identifying the point of obstruction and, in association with the endoscopic procedures, can allow definitive extraction of the foreign body, where possible. In our case, the colonoscopy was successful in completely removing the bezoar.

Sometimes the poor general conditions of the patient do not allow a time-consuming procedure but an emergency laparotomy, and so the minimally-invasive treatment is not indicated (6).

In adults, endoscopic treatments of bezoars are reported, but only for gastric bezoars and include the use of alligator forceps, lithotripters, needle cutters, snares, and holmium:yttrium aluminum garnet laser lithotripsy (7).

At now, no reports of endoscopic treatment of colonic bezoars are reported, due to the extreme rarity of these lesions, the size as limiting factor, and the fear of spillage and peritoneal contamination if the mass would incidentally break and perforate the viscera.

Endoscopic approach gives the advantage of a reduced trauma, faster recovery, fewer complications, and low cost. Recently Huang et al (8) described a patient with a giant hard gastric bezoar that was successfully removed by combined dual knife–electric snare treatment, without any complications.

## CONCLUSIONS

Laparo-endoscopic management of small/large bowel bezoars was feasible and effective in our case. Psychologic referral, follow-up, and support remain mandatory to prevent recurrence. This is, to our knowledge, the first-ever reported case of a giant colonic trichobezoar with near-total colonic obstruction.
